# Percutaneous internal ring suturing versus conventional laparoscopic repair of congenital inguinal hernia: a randomized controlled trial

**DOI:** 10.1007/s00464-025-12309-9

**Published:** 2025-11-17

**Authors:** Israa Saad, Mostafa Zain, Ahmed Khairi, Mohamed Abouheba, Baher Louka

**Affiliations:** Alexandria Faculty of Medicine, University of Alexandria, Alexandria, 21615 Egypt

**Keywords:** Congenital inguinal hernia, Laparoscopic hernia repair, Percutaneous internal ring suturing, Pediatric surgery, Minimally invasive surgery

## Abstract

**Background:**

Laparoscopic repair of congenital inguinal hernia (CIH) has evolved with techniques to minimize invasiveness while ensuring efficacy. This study compares percutaneous internal ring suturing (PIRS) with laparoscopic disconnection of the hernial sac and internal ring narrowing regarding feasibility, safety, operative time, cosmetic outcome, and recurrence rate.

**Methods:**

A prospective randomized study was conducted on 109 male patients with 120 CIHs. Patients were allocated into two groups: Group A (laparoscopic disconnection and internal ring narrowing) and Group B (PIRS). Perioperative parameters, postoperative complications, recurrence rates, and parental satisfaction with cosmetic results were assessed.

**Results:**

The mean operative time was significantly shorter in group B (8.5 ± 3.9 min for unilateral cases) compared to group A (40.8 ± 9.9 min) (*p* < 0.001). Postoperative spermatic cord edema occurred in 19.6% of Group A and 1.9% of Group B (*p* < 0.05). Recurrence rates were 5.4% in Group A and 1.9% in Group B (*p* > 0.05). Parental satisfaction with scarring was significantly higher in group B (*p* < 0.05).

**Conclusion:**

In this randomized trial, PIRS resulted in shorter operative time, lower incidence of spermatic cord edema, and higher parental satisfaction compared with conventional laparoscopic repair in pediatric patients with congenital inguinal hernia.

**Level of Evidence:**

II (Prospective randomized study).

## Introduction

Congenital inguinal hernia (CIH) is one of the most prevalent surgical conditions in pediatric patients, with an incidence ranging from 0.8% to 4.4% [1]. It accounts for more than 15% of pediatric surgical procedures and is generally considered a well-tolerated operation with a low risk of significant complications [2]. Traditionally, CIH repair has been performed via an open inguinal approach, which remains the most widely utilized technique worldwide [3]. However, during the last three decades, laparoscopic techniques have gained significant traction, offering advantages, such as enhanced cosmesis, reduced postoperative pain, expedited recovery, and the ability to assess the contralateral inguinal ring [4]. Despite these benefits, the routine adoption of laparoscopic CIH repair has been hindered by concerns related to increased costs, an extended learning curve, and a longer operative duration [5]. Nevertheless, a recent cross-sectional survey of pediatric surgeons in the USA demonstrated a shift in clinical practice patterns. Although approximately 75% of respondents reported having received formal training in the open technique, more than two-thirds indicated that they currently incorporate laparoscopic inguinal hernia repair into their routine practice [6].

A variety of laparoscopic techniques for CIH repair have been described, encompassing intracorporeal and extracorporeal approaches. Among these, laparoscopic disconnection of the hernial sac with internal ring narrowing has been developed to replicate the principles of open hernia repair while potentially reducing recurrence rates [5]. Conversely, percutaneous internal ring suturing (PIRS) represents a less invasive alternative that requires only a single umbilical port and utilizes a percutaneous needle to encircle the internal ring, thereby eliminating the hernial defect with minimal scarring [7]. The cosmetic superiority of PIRS, coupled with its reduced invasiveness, has made it an attractive option in pediatric surgery [7].

Given the evolving landscape of minimally invasive techniques for CIH repair, this study aimed to compare the short-term outcomes of PIRS versus laparoscopic disconnection of the hernial sac with internal ring narrowing in children regarding feasibility, safety, operative details, cosmetic outcome, recurrence rate, and postoperative complications.

## Patients and methods

This prospective randomized clinical study was conducted in the Pediatric Surgery Unit at Alexandria University Children’s Hospital between May 2022 and May 2024. The study enrolled 109 male patients with a total of 120 CIHs, ranging in age from 6 months to 5 years. Exclusion criteria included incarcerated or recurrent CIH, associated ipsilateral undescended testis or hydrocele, and chronic comorbidities such as congenital heart disease or severe respiratory conditions. Patients were randomized into two groups using block randomization (ABAB); group A who underwent laparoscopic disconnection of the hernia sac and narrowing of the internal inguinal ring (IIR), and group B who were treated with PIRS.

The sample size for this study was calculated By SPSS program version 28 [8], A minimal total sample size of (114) pediatric male patients undergoing Congenital Inguinal Hernia repair: (57) per group was needed to detect an assumed difference of (16.6%) in the percentage of hernia recurrence between a group of patients receiving Percutaneous Internal Ring Suturing vs another group receiving Conventional Laparoscopic Repair with an assumed recurrence rate of (18.6%, 2%), respectively [9, 10], using chi-square test with a significance level of (0.05), and 80% power after adding 10% loss to follow-up.

This research was performed in accordance with the Declaration of Helsinki, and it was approved by the ethical committee of Alexandria Faculty of Medicine under No: 0107373, and registered on clinicaltrials.gov under ID: NCT06856304.Written informed consent to participate in this study was provided by the participants’ legal guardian/next of kin.

### Operative technique

Patients were placed in the supine position. A 5-mm umbilical trocar was inserted using either the Veress needle or the open (Hasson) technique, according to the surgeon’s preference, and pneumoperitoneum was established with carbon dioxide at 8–10 mmHg. Operative time was recorded from this point, a standardized step common to both approaches, to minimize potential confounding related to the method of abdominal access. A 30-degree laparoscope was then introduced through the umbilical port. Hernial contents were manually reduced externally, and the internal rings were identified bilaterally to confirm the diagnosis (Table 1).

**Table 1:** Comparative outcomes of laparoscopic disconnection with internal ring narrowing (Group A) versus Percutaneous Internal Ring Suturing (PIRS) (Group B)

	Group A (*n* = 56) No. (%)	Group B (*n* = 53) No. (%)	Statistics	*p*
Age (months) Min.–Max Mean ± SD Median (IQR)	6.0–60.0 21.59 ± 16.28 15.0 (9.0–33.0)	6.0–60.0 25.82 ± 17.81 24.0 (10.0–48.0)	Independent *t*-test	> 0.05
Site Right Left Bilateral	36 (64.3) 16 (28.6) 4 (7.1)	31 (58.5) 15 (28.3) 7 (13.2)	*χ*^2^ 1.142	0.565
Operative time (min.) Unilateral cases Min.–Max Mean ± SD Bilateral cases Min.–Max Mean ± SD	20.0–65.0 40.84 ± 9.74 45.0–70.0 58.75 ± 11.086	3.0–20.0 8.48 ± 3.94 15.0–35.0 21.42 ± 8.01	Independent *t*-test	< 0.001*
Complications Edema Recurrence	11 (19.6) 3 (5.4)	1 (1.9) 1 (1.9)	*χ*^2^ 9.25 1.03	0.002 0.3
Parental satisfaction (scarring)	8.7 ± 1.2	9.3 ± 0.9	Independent *t* test	< 0.05*

*IQR* Inter quartile range, *SD* Standard deviation, *χ*^2^ Chi-square test, *p p* value for comparing the two studied groups

^*^Statistically significant at *p* ≤ 0.05

**Fig. 1:**
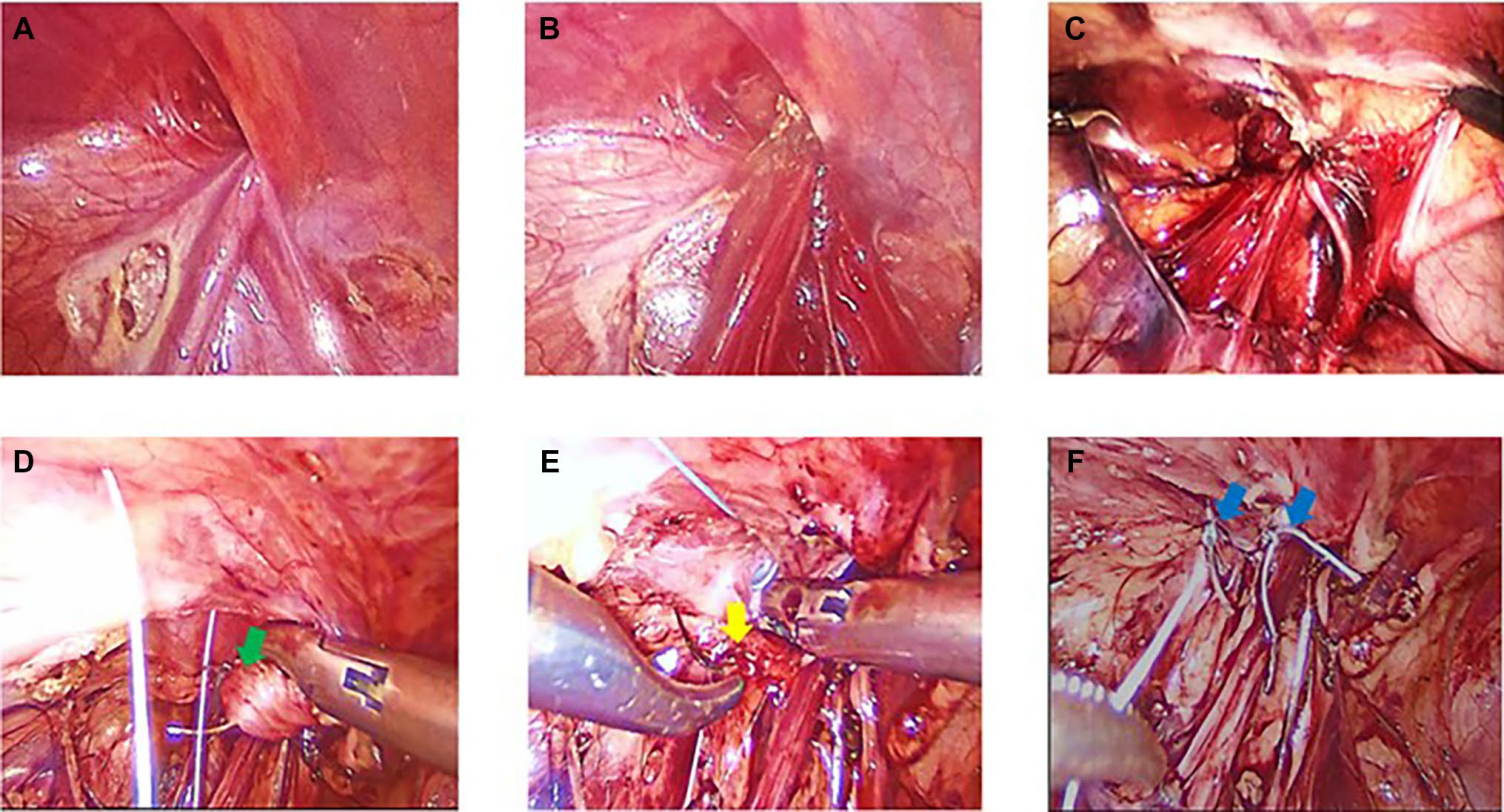
Operative images illustrating the key steps in disconnecting the hernial sac and narrowing the internal ring. **A** Two small openings in the peritoneum were created on either side of the vas deferens and vessels using hook diathermy. **B**, **C** Complete disconnection of the peritoneum was achieved over the vas deferens and vessels. **D**, **E** A suture was placed between the transverse arch of fascia transversalis (green arrow) and the iliopubic tract (yellow arrow). **F** Two sutures were positioned to narrow the internal ring lateral to the vas deferens and vessels (blue arrows)

In group A (Laparoscopic Disconnection and IIR Narrowing), two additional 3-mm trocars were inserted at the mid-clavicular lines at the umbilical level to maintain triangulation. The patient was then placed in Trendelenburg’s position. The open IIR was identified, and traction was applied to the hernia sac using a grasper. The edges of the sac were cauterized using hook diathermy, starting laterally and progressing medially, where the vas deferens and vessels were carefully dissected away from the sac without diathermy. The distal portion of the sac was dropped into the inguinal canal. The internal ring was narrowed by approximating the iliopubic tract (IPT) to the transverse arch of the transversus abdominis (TATA) using interrupted Vicryl 3/0 sutures placed lateral to the cord structures. Care was taken to avoid excessive tightness, with 1–2 sutures typically sufficient to achieve adequate narrowing (Fig. 1).

**Fig. 2:**
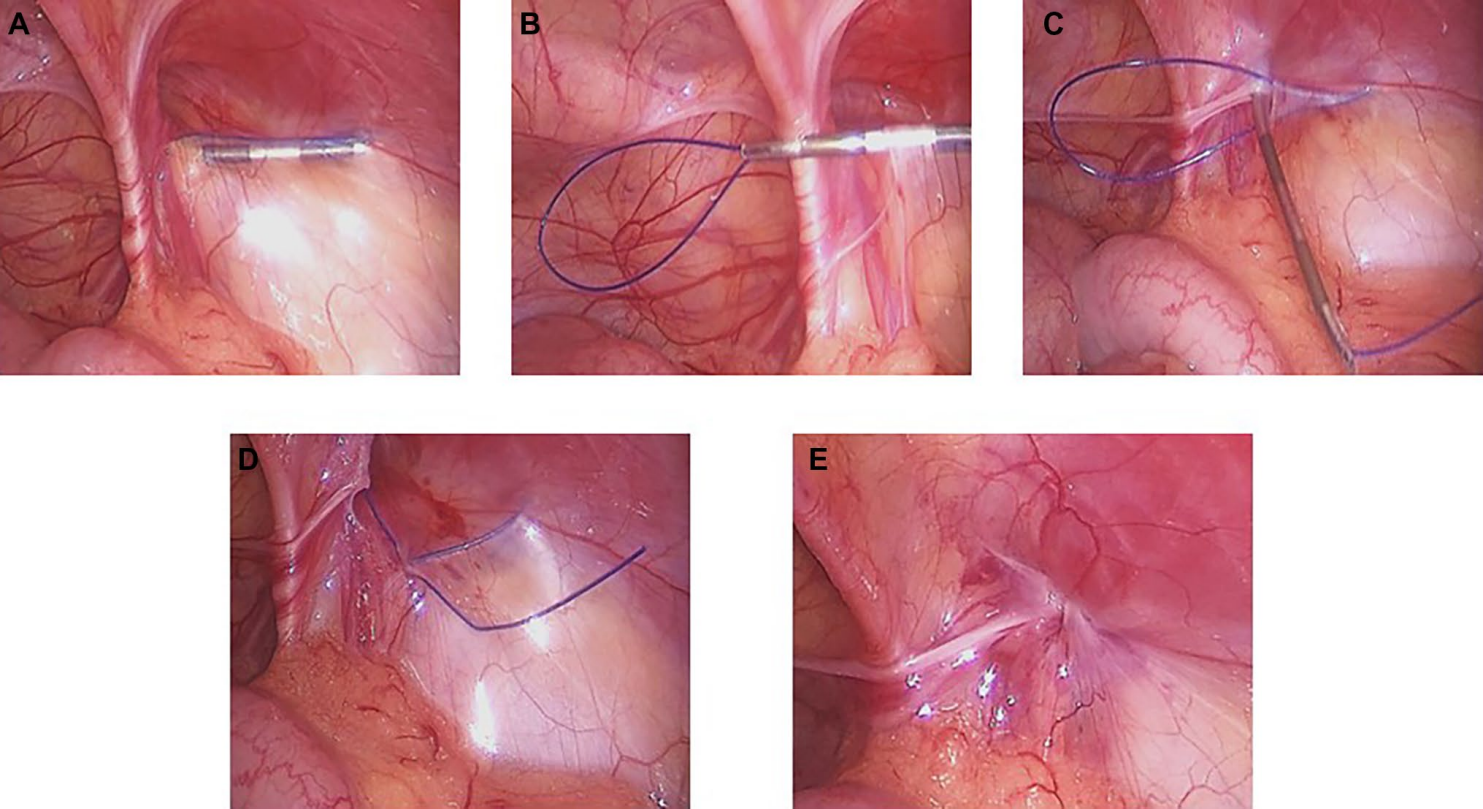
Operative images demonstrating the main steps of the PIRS technique. **A** The needle tip is passed under the peritoneum, involving half of the internal ring. **B** A thread is inserted through the needle, forming a loop. **C** The needle encircles the remaining half of the internal ring, avoiding the area over the vas deferens and vessels. **D** Another thread is passed through the barrel of the needle into the thread loop, and the needle is withdrawn. **E** The knot is tied to close the internal ring

In group B (PIRS), an 18-gauge injection needle was bent into a gentle curve and threaded with a looped non-absorbable 2–0 monofilament suture. A single umbilical trocar was inserted through a supraumbilical incision. The position of the IIR was identified by applying external pressure to the inguinal region using the tip of a forceps. Under laparoscopic guidance, the prepared needle was introduced through a skin puncture at the lateral edge of the internal ring. The suture was passed under the peritoneum, encircling the lateral half of the IIR, and a loop was formed within the abdominal cavity. The needle was withdrawn, leaving the loop in place. The loop was then externalized, capturing the free end of the suture, which was tied to close the internal ring. The knot was secured subcutaneously, and the umbilical wound was closed with absorbable sutures. The inguinal puncture site was left uncovered, with only a sterile dressing applied. (Fig. 2).

All cases in each group were performed exclusively by the same experienced consultant pediatric surgeons. This distribution was maintained throughout the study to standardize the technique within each group.

### Postoperative care and follow-up

In both groups, oral fluids were initiated 2 h after full recovery from anesthesia, and all patients were discharged on the same day. Follow-up evaluations were conducted at 48 h, 1 week, 1 month, and 6 months postoperatively (or contacted by phone for the late 3, 6-month, and 1-year follow-ups). Assessments included monitoring for postoperative edema, hernia recurrence, testicular atrophy, and parental satisfaction with scarring. Scarring was evaluated using a 10-point scale, with scores of 1–3 considered poor, 4–7 fair, and 8–10 good. The primary outcome was hernia recurrence at one year postoperatively, while secondary outcomes included operative time, efficacy, safety, and postoperative complications. The study protocol was approved by the local ethics committee of the Faculty of Medicine at Alexandria University, and written informed consent was obtained from all parents or legal guardians prior to participation.

### Statistical analysis of the data

Data were fed to the computer and analyzed using IBM SPSS software package version 20.0 (Armonk, NY: IBM Corp, released in 2011). Qualitative data were described using numbers and percentages. The Kolmogorov–Smirnov test was used to verify the normality of distribution. Quantitative data were described using range (minimum and maximum), mean, standard deviation, median, and interquartile range (IQR). The significance of the results obtained was judged at the 5% level.

The tests used were

Chi-square test: For categorical variables, to compare different groups.

Fisher’s Exact: Correction for chi-square when more than 20% of the cells have an expected count less than 5.

Student *t* test: For normally distributed quantitative variables, to compare two studied groups.

4—Mann–Whitney test: For abnormally distributed quantitative variables, to compare between two studied groups.

## Results

This study involved 109 male patients with a total of 120 CIHs, who were randomized into two groups using block randomization (ABAB). Group A comprised 56 infants and children with 60 hernias, while Group B included 53 infants and children with 60 hernias. The median age was 20.5 months in Group A and 23 months in Group B, with no significant difference in age distribution between the two groups (*p* > 0.05). In Group A, 64.3% of cases are presented with right-sided inguinal hernias, 28.6% with left-sided hernias, and 7.1% with bilateral hernias. In Group B, 58.5% of cases had right-sided hernias, 28.3% had left-sided hernias, and 13.2% were bilateral. There was no significant difference in the distribution of hernia laterality between the two groups (*p* > 0.05).

The operative time, measured after umbilical trocar insertion to abdominal deflation, ranged from 25 to 64 min in Group A, with a mean duration of 40.8 ± 9.9 min for unilateral cases and 83.3 ± 13.8 min for bilateral cases. In Group B, the operative time was significantly shorter, ranging from 3 to 20 min, with a mean duration of 8.5 ± 3.9 min for unilateral cases and 19.3 ± 3.8 min for bilateral cases (*p* < 0.001). None of the cases required conversion to open surgery.

Intraoperative complications were minimal, with one case of preperitoneal bleeding in Group A occurring during peritoneal incision and one case in Group B resulting from repeated PIRS attempts. Both cases were managed conservatively, with spontaneous cessation of bleeding. No injuries to the vas deferens or vessels were reported. All patients achieved full recovery, resumed oral intake within 2 h postoperatively, and were discharged on the same day.

Postoperative follow-up revealed spermatic cord edema in 11 cases (19.6%) in Group A and one case (1.9%) in Group B. The edema resolved spontaneously within one week in all cases, with the incidence being significantly higher in Group A compared to Group B (*p* < 0.05). Recurrence occurred in 3 out of 60 hernias (5%) in Group A and 1 out of 60 hernias (1.7%) in Group B. Although the recurrence rate was lower in Group B, the difference was not statistically significant (*p* > 0.05). All recurrent cases were managed laparoscopically. In Group A, recurrence was attributed to a large, skipped area over the vas deferens and vessels, which was addressed by completing the peritoneal disconnection. In Group B, recurrence resulted from a slipped ligature, which was managed by disconnecting the peritoneum at the internal ring level and narrowing the ring with sutures. No cases of postoperative hydrocele or testicular atrophy were observed in either group.

Parental satisfaction with scarring, assessed using a 10-point scale, revealed a median score of 8.0 (7.0–9.0) in Group A and 9.0 (8.0–9.0) in Group B, with satisfaction being significantly higher in Group B (*p* < 0.05). Additionally, the mean hospital stay was comparable between the two groups, with all patients discharged on the same day. The overall success rate, defined as the absence of recurrence and complications, was 95%in Group A and 98.3% in Group B, with no significant difference between the groups (*p* > 0.05).

## Discussion

The evolution of laparoscopic techniques in pediatric inguinal hernia repair has undergone significant advancements since its introduction in the early 1990s. Initially, laparoscopy was primarily used for diagnosing contralateral hernias, given the high incidence of bilateral inguinal hernias in pediatric patients [1]. The transition from diagnostic laparoscopy to a therapeutic approach began with Schier and El-Gohary, who pioneered laparoscopic hernia repair in female patients, avoiding the risk of injury to the vas deferens and testicular vessels in males [11, 12]. Montupet and Esposito were among the first to use laparoscopy in male CIH, using intracorporeal purse-string closure of the internal ring [13]. Since then, several modifications have been developed, driven by the desire to minimize invasiveness, reduce complications, and improve cosmetic outcomes, all of which are particularly important in pediatric patients. Whereas early techniques involved intracorporeal approaches such as laparoscopic technology and techniques advanced, extracorporeal approaches were developed, such as PIRS, which offers several advantages, including reduced operative time, fewer ports, and minimal scarring [7].

Early experiences with laparoscopic repair of CIH, particularly in male patients, were associated with elevated recurrence rates when the peritoneum was closed intact. This was predominantly attributed to the technical challenges inherent in achieving a secure closure over the vas deferens and testicular vessels, frequently resulting in residual “skip areas” that compromised the integrity of the repair [14, 15]. In an effort to mitigate this issue, the concept of deliberately disrupting peritoneal continuity at the internal ring was introduced to induce local fibrosis and enhance closure stability. Several studies demonstrated the safety of sutureless repair using sac disconnection alone, especially in cases with small to moderate internal ring diameters, reporting low to negligible recurrence rates [16–19]. In contrast, larger defects were often managed with additional ring closure using a purse-string suture. While effective, this technique carries a potential risk of injury to the spermatic cord structures [20–22].

At our institution, based on cumulative clinical experience and the existing body of literature, we adopted a hybrid technique involving sac disconnection with internal ring narrowing by approximating the IPT to the TATA using one or two interrupted sutures. This approach permits controlled narrowing of the internal ring without complete closure, thereby reducing the risk of injury or entrapment of the spermatic cord structures. This method of tissue repair has been well established in open hernia surgery and has more recently been adapted to the laparoscopic setting, particularly for recurrent cases [23–26].

Although our institutional protocol adheres to the pediatric hernia classification and tailored management strategy described by Shehata et al. [27], which advocates for selective tissue repair only in cases with demonstrably wide internal rings, we deliberately designed this study to compare PIRS with a standardized laparoscopic approach that includes routine internal ring narrowing, regardless of IIR diameter. This decision was made to ensure methodological consistency and to establish a uniform comparator group. By controlling for variability in surgical decision-making, this design allows for a clear evaluation of outcomes between two distinct and reproducible techniques. We consider this trial a foundational step that provides essential comparative data. In subsequent studies, we plan to explore a more individualized laparoscopic strategy, where the choice to perform tissue repair is guided by intraoperative measurement of IIR diameter. We believe this stepwise, evidence-based approach will support refinement of surgical management by balancing the potential benefits of tailored repair with the risks of unnecessary dissection in patients with anatomically narrow rings.

In our study, we included only male patients aged 6 months to 5 years to create a homogeneous and comparable group. The age range was chosen based on the high incidence of CIH in early childhood, as well as the need to minimize variability due to differences in anatomic size and tissue characteristics [2]. Studies such as those by Ein et al. and Lao et al. have demonstrated that most pediatric hernias present within the first few years of life, with peak incidence in infancy [2, 3]. By focusing on this age group, we ensured that the surgical techniques could be standardized, and the results would be more generalizable to the population most affected by CIH.

The decision to include only male patients was based on the anatomic complexity of male inguinal hernia repair. The presence of the vas deferens and testicular vessels in males makes the procedure more challenging than in females, as these structures are at risk of injury during both intracorporeal and extracorporeal approaches [14]. Previous studies, including those by Schier and El-Gohary, initially focused on female patients to avoid these complications in the early development of laparoscopic techniques [12, 28]. However, as laparoscopic skills and instrumentation improved, researchers began evaluating male patients. Becmeur et al. studied 67 males and 15 females, while Chinnaswamy et al. included 56 males and only 8 females [12, 29]. By focusing exclusively on male patients, our study provides valuable insights into the efficacy and safety of laparoscopic techniques in a more challenging anatomic context.

One of the major disadvantages of laparoscopic hernia repair compared to open surgery is its longer operative time, particularly for intracorporeal techniques requiring suturing [30]. In our study, the mean operative time for unilateral cases in the intracorporeal group (Group A) was 40.8 ± 9.9 min, while bilateral cases required 83.3 ± 13.8 min. In contrast, the PIRS technique (Group B) was significantly faster, with a mean operative time of 8.5 ± 3.9 min for unilateral cases and 19.3 ± 3.8 min for bilateral cases (*p* < 0.001).

Our findings align with those of Wang et al., who compared laparoscopic intracorporeal suturing (LIS) to laparoscopic percutaneous extracorporeal closure (LPEC) using a Reverdin needle. They reported significantly shorter operative times in the LPEC group (15.76 ± 5.35 min for unilateral and 21.56 ± 5.7 min for bilateral cases) compared to LIS (19 ± 5.71 min and 26.38 ± 6.94 min, respectively) [31]. Although our PIRS times were shorter than Wang’s LPEC, our intracorporeal method took longer, likely due to the additional step of disconnecting the hernia sac.

Shalaby et al. also demonstrated that extracorporeal techniques significantly reduce operative time compared to intracorporeal repairs [32]. Their study, which used extracorporeal suturing with a Reverdin needle, showed a mean operative time of 13.5 ± 2.3 min for unilateral cases and 22.8 ± 4.1 min for bilateral cases, like our findings in the PIRS group. These results suggest that PIRS offers a significant advantage in terms of operative efficiency, which is particularly important in pediatric surgery where shorter operative times are associated with reduced anesthesia-related risks.

The use of different suture materials between the study groups reflected the standardized protocols inherent to each technique and was applied consistently; however, their potential impact on outcomes cannot be entirely excluded. In Group A, absorbable sutures were utilized based on the rationale that, following deperitonealization and exposure of the raw muscular arch, wound healing is primarily mediated by scar tissue formation rather than long-term retention of foreign material [14]. In contrast, Group B employed non-absorbable sutures to ensure secure closure of the internal inguinal ring, as this technique does not involve separation of the peritoneum at the level of the internal ring. Thus, the integrity of the closure in Group B relies predominantly on the suture material itself [7, 16, 33].

Intraoperative complications were minimal in both groups. One case of preperitoneal bleeding occurred in Group A while incising the peritoneum, and another in Group B due to repeated trials of PIRS. Both were managed conservatively without conversion to open surgery. Importantly, we observed no cases of vas deferens or vascular injury, which is a significant concern in male laparoscopic hernia repair [14]. This is consistent with the findings of Patkowski et al., who reported no major intraoperative complications in their study of PIRS [7].

Postoperatively, spermatic cord edema was significantly higher in the intracorporeal group (19.6%) compared to the PIRS group (1.9%) (*p* < 0.05). Spermatic cord edema was assessed clinically by comparison with the contralateral side, documenting visible swelling and palpable thickening along the cord. Although this evaluation is inherently subjective and the edema was transient, self-limiting, and resolved spontaneously in all cases without functional or cosmetic sequelae, its documentation remains noteworthy, as such swelling may generate considerable parental concern when misinterpreted as hernia recurrence. The lower incidence of postoperative edema in the PIRS group is likely due to the minimal manipulation of the spermatic cord structures, which reduces trauma to the surrounding tissues. This finding is consistent with the study by Chinnaswamy et al., where scrotal swelling occurred in only one out of 64 patients undergoing laparoscopic ring closure [29]. Similarly, Oshieba et al. reported a 10% incidence of scrotal swelling, which resolved spontaneously [30].

Recurrence occurred in 3 out of 60 hernias (5.4%) in Group A and 1 out of 60 hernias (1.9%) in Group B. Although recurrence was higher in the intracorporeal group, the difference was not statistically significant (*p* > 0.05). Our recurrence rate in the intracorporeal group is slightly higher than the 4% recurrence rate reported by Shalaby et al. for intracorporeal suturing but comparable to their extracorporeal method, which had a recurrence rate of 1.3% [32].

Patkowski et al. reported a 2.8% recurrence rate for PIRS, aligning closely with our findings [7]. Giseke et al., in a study of 385 children, found a recurrence rate of 1.04%, reinforcing the reliability of the PIRS approach [34]. The slightly higher recurrence rate in our intracorporeal group may be attributed to the technical challenges associated with intracorporeal suturing, particularly in ensuring complete closure of the internal ring without leaving gaps that could lead to recurrence.

Parental satisfaction with scarring was significantly higher in the PIRS group 9.0 (8.0–9.0) compared to the intracorporeal group 8.0 (7.0–9.0) (*p* < 0.05). The superior cosmetic outcome in PIRS is expected, as the technique requires only a single umbilical port and a small skin puncture, resulting in minimal visible scarring. This finding is consistent with previous studies that have reported higher cosmetic satisfaction with PIRS [7, 35]. For example, Patkowski et al. noted that parents were highly satisfied with the cosmetic outcome of PIRS, with most reporting that the scars were barely noticeable [7]. The cosmetic advantage of PIRS is particularly important in pediatric surgery, where the psychological impact of scarring can be significant. Parents often express concern about the long-term appearance of surgical scars, and the ability to offer a technique that minimizes visible scarring can greatly enhance their overall satisfaction with the surgical outcome. In addition to the cosmetic benefits, the shorter operative times and reduced postoperative complications associated with PIRS contribute to higher parental satisfaction [36, 37].

This study has some limitations. First, although randomization and standardized protocols were applied to reduce bias, all procedures were performed by experienced consultant pediatric surgeons, whose technical expertise and individual variations may have introduced performance bias. In addition, different suture materials were used between the two techniques, which could represent a potential confounding factor. The relatively short follow-up period of one year may not be sufficient to capture late recurrences. Finally, the study did not stratify outcomes according to internal ring diameter, which may be an important factor in tailoring surgical management. Future studies with larger cohorts, longer follow-up, and stratification by internal ring diameter are recommended to validate these findings and optimize technique selection.

## Conclusion

In this randomized trial, PIRS resulted in shorter operative time, lower incidence of spermatic cord edema, and higher parental satisfaction compared with conventional laparoscopic repair in pediatric patients with congenital inguinal hernia.

## Data Availability

The datasets used and/or analyzed during the current study are available from the corresponding author on reasonable request.

## List of Abbreviations

CIH: Congenital inguinal hernia
PIRS: Percutaneous internal ring suturing
IIR: Internal inguinal ring
LPEC: Laparoscopic percutaneous extracorporeal closure
LIS: Laparoscopic intracorporeal suturing
IPT: Iliopubic tract
TATA: Transverse arch of the transversus abdominis
